# Pyrrolizidine Alkaloids: Chemistry, Pharmacology, Toxicology and Food Safety

**DOI:** 10.3390/ijms19061668

**Published:** 2018-06-05

**Authors:** Rute Moreira, David M. Pereira, Patrícia Valentão, Paula B. Andrade

**Affiliations:** REQUIMTE/LAQV, Laboratório de Farmacognosia, Departamento de Química, Faculdade de Farmácia, Universidade do Porto, R. Jorge Viterbo Ferreira, nº 228, 4050-313 Porto, Portugal; rutemartinsmoreira@hotmail.com (R.M.); valentao@ff.up.pt (P.V.)

**Keywords:** pyrrolizidine alkaloids, chemistry, toxicity, pharmacological properties, food safety

## Abstract

Pyrrolizidine alkaloids (PA) are widely distributed in plants throughout the world, frequently in species relevant for human consumption. Apart from the toxicity that these molecules can cause in humans and livestock, PA are also known for their wide range of pharmacological properties, which can be exploited in drug discovery programs. In this work we review the current body of knowledge regarding the chemistry, toxicology, pharmacology and food safety of PA.

## 1. Introduction

Alkaloids are a diverse group of amino acid-derived and nitrogen-bearing molecules that display a wide range of roles in nature, where they occur in plants, microorganisms or animals [1]. In plants, alkaloids can be found in the form of salts of organic acids, mainly malate, acetate and citrate, or combined with other molecules, such as tannins [1]. Most alkaloids display basic properties and present a lipophilic character, being soluble in apolar organic solvents and alcohol [1].

Several alkaloids are known for their remarkable biological properties, which can be either marked toxicity or potent pharmacological capacity [2]. The class of alkaloids has a long history of use, both lawful and illicit, as pharmaceuticals, stimulants and narcotics [3].

Within the many known families of alkaloids, pyrrolizidine alkaloids (PA) have been receiving increasing attention due to their occurrence in several species relevant for human and animal nutrition, as well as for their toxicological and pharmacological properties. The increasing awareness of PA contamination in all sorts of foodstuff worldwide justifies the interest and concern around this topic. Although in most cases their levels are insufficient to cause acute poisoning, they are frequently consumed in quantities that exceed the maximum daily intake suggested by authorities, which can be a contributory factor to chronic diseases.

This work reviews the available information on PA, mainly regarding their chemistry, toxic and pharmacological properties, and food safety.

## 2. The Chemistry of PA

**Figure 1:**
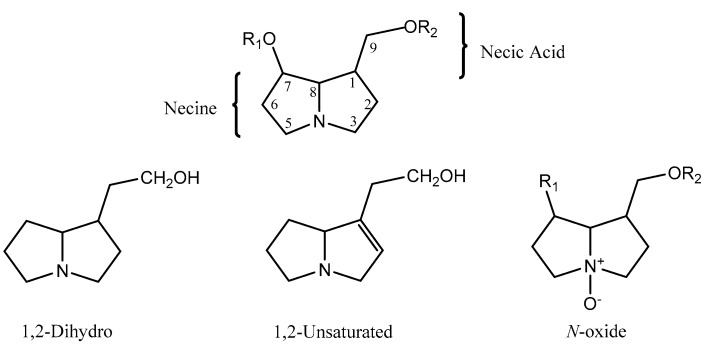
Structure of a PA and its different forms. R_1_ and R_2_ correspond to different necic acids.

PA are a group of alkaloids derived from ornithine that are distributed in plants of certain taxa, being also found in insects that uptake them for defense against predators [1,4]. They rarely occur in the free form as a pyrrolizidine base, being instead found as esters (mono-, di- or macrocyclic diesters) formed by a necine base (amino alcohols) and one or more necic acids (mono- or dicarboxylic aliphatic acids), which are responsible for their structural diversity [1,5]. They are usually found in the form of tertiary bases or pyrrolizidine alkaloids *N*-oxides (PANO) (Figure 1) [1,5,6].

Amino alcohols, or necines, are derived from pyrrolizidine. The pyrrolizidine core, comprising two saturated five-membered rings with a nitrogen atom between them, sometimes displays a double bond in the 1,2 position, which frequently results in enhanced toxicity [7]. They can also have a single alcohol at C-1, a second alcohol in position C-7 (di-hydroxylated) and less often a third in C-2 or C-6 (tri-hydroxylated) [8,9,10]. Esterification can take place at C-7 and/or C-9 positions [9].

**Figure 2:**
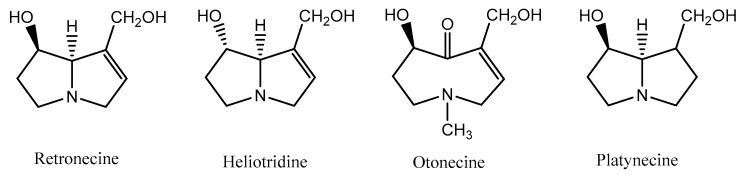
Groups of PA, according to the necine base.

According to the structure of the necine base, PA may be sorted into four groups: retronecine-, heliotridine-, otonecine- and platynecine-types (Figure 2) [11]. Retronecine-, otonecine- and heliotridine-types are unsaturated bases, while platynecine-type is saturated [12,13]. From a structural point of view, otonecine is the most distinct among all types, since it is oxidized at C-8 and displays a monocyclic ring, thus diverging from the other groups, which display a bicyclic ring [8,10,14]. Retronecine and heliotridine are diastereomers, with distinct orientation at position C-7 [15].

**Figure 3:**
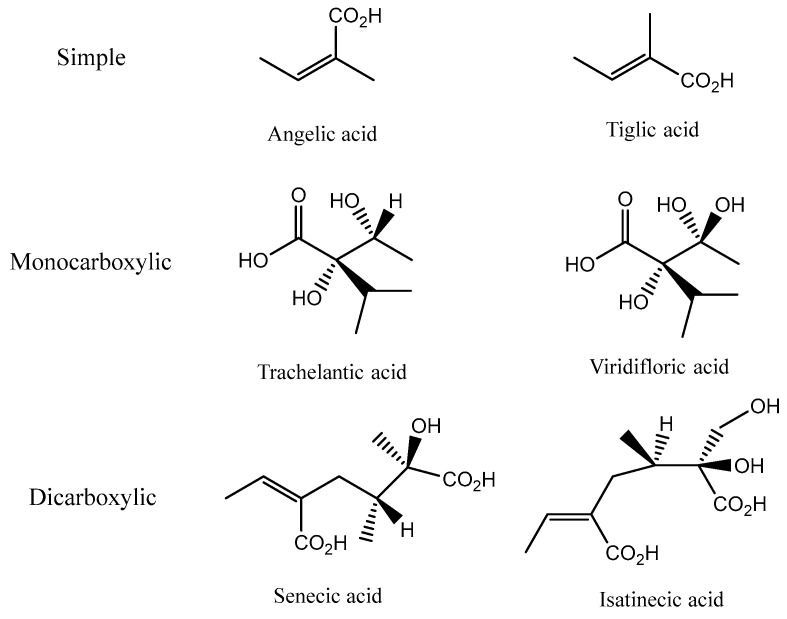
Chemical structures of necic acids.

Necic acids are aliphatic carboxylic acids that can be simple (angelic and tiglic acids), monocarboxylic acids with hydroxyisopropylbutanoic structures at C-7 (trachelantic and viridifloric acids) or dicarboxylic acids at C-8 or at C-10 (senecic and isatinecic acids) (Figure 3) [1].

**Figure 4:**
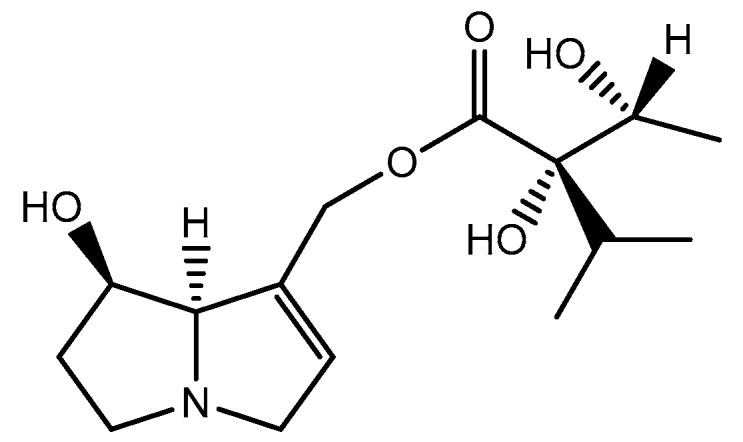
Chemical structure of intermedine, an ester of monocarboxylic acid.

**Figure 5:**
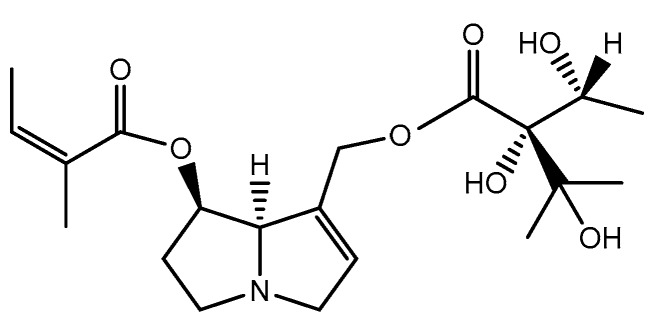
Chemical structure of echimidine, a diester of monocarboxylic acid.

**Figure 6:**
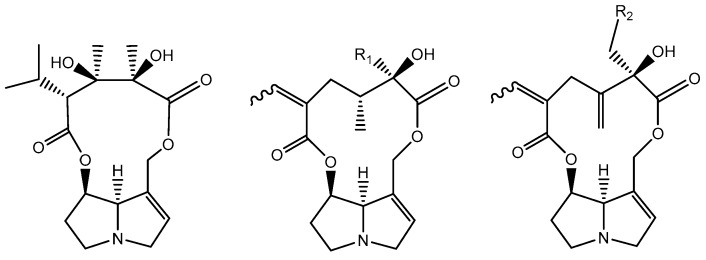
Chemical structure of tricodesmine, senecionine (R_1_ = H)/retrorsine (R_1_ = OH) and seneciphylline (R_2_ = H)/riddeline (R_2_ = OH), macrocylic diesters.

The combination of the above-mentioned structures results in mono- or di-esters. Within the monocarboxylic acids, characteristic of the Boraginaceae family, some have a hydroxyl group at C-9 esterified by a hydroxyisopropylbutanoic acid, such as intermedine (Figure 4) [1]. In cases where there is a second necic acid, it usually occurs in the hydroxyl group of C-7, in the form of angelic acid or tiglic acid, as in echimidine (Figure 5) [1]. Macrocyclic diesters, characteristic from Asteraceae family, have also been described, which correspond to C-7 and C-9 esterified by a dicarboxylic acid (Figure 6) [1]. Unusually, necines may be esterified with aromatic or arylalkyl acids [1].

**Figure 7:**
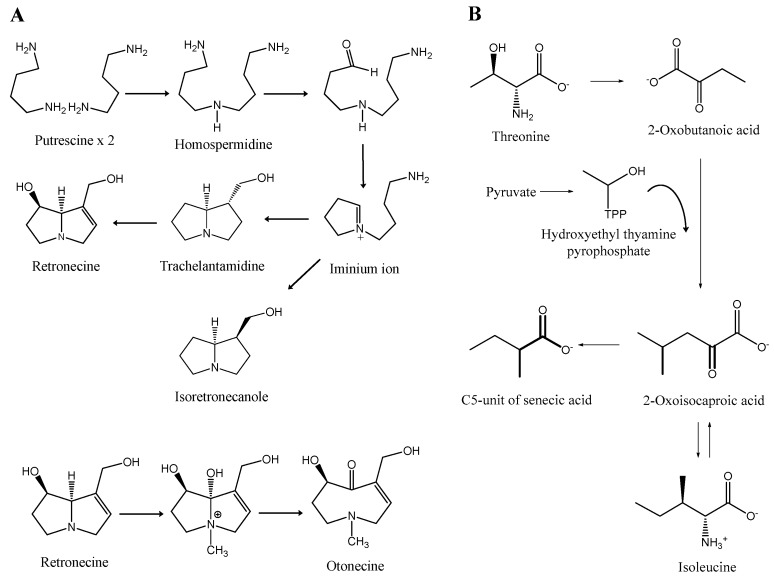
Biosynthesis of necines (**A**) and of senecic acid (**B**). Adapted from [1,16,20,24].

According to the most widely accepted pathway, the biosynthesis of the pyrrolizidine core begins with a NAD^+^-dependent condensation of two molecules of putrescine. It should be highlighted that this initial step is disputed by some authors, which advocate the involvement of one molecule of putrescine and one molecule of spermidine, the latter providing the aminobutyl group [16,17,18]. Interestingly, it could be the case that both theories are correct, as suggested by the finding that bacterial homospermidine synthase is able to accept either putrescine and spermidine as a substrate [19]. Regardless of the initial step, in both cases the reaction is catalyzed by homospermidine synthase and the result is the symmetrical intermediate homospermidine [1]. Subsequently, homospermidine is cyclized to the corresponding iminium ion, which is reduced and cyclized to trachelanthamidine and isoretronecanole (Figure 7A) [20].

Regarding necic acids, they are mostly derived from l-valine, l-leucine, l-isoleucine and l-threonine [20].

The formation of monocarboxylic acids with five carbon atoms, such as angelic, tiglic and sarracinic acid, takes place through the metabolization of threonine, which in turn proceeds from via α-ketobutyric acid, also called 2-oxobutanoic acid. The interaction between this compound and pyruvate yields isoleucine [20].

With respect to senecioic, viridifloric and trachelanthic acids, the precursor involved is valine, which suffers a conversion into these necic acids, via an acyloin reaction with activated acetaldehyde [20].

In the case of dicarboxylic acids such as senecic acid, with ten carbon atoms (Figure 7B), cyclization of the open-chain monocarboxylic acid diesters takes place [20]. The biosynthesis occurs in the roots, where they are formed as PANO [21]. Afterwards, due to their high solubility in water, they are easily transported to the aerial parts so they can be stored in cell vacuoles [21].

Concerning the chemical synthesis, there has been an immense amount of research conducted on the partial and total synthesis of numerous naturally-occurring PA and related non-natural analogues [22,23].

## 3. Biological Activity of PA

### 3.1. Pharmacokinetics

Concerning PA pharmacokinetics, after oral ingestion these compounds are absorbed from the gastrointestinal tract [25]. Most of them, around 80%, are excreted in urine, feces and milk, a few being able to pass the placenta due to their high lipophilicity [25,26,27]. Bioactivation occurs mostly in the liver and, for this reason, this organ is the most affected by toxicity [28]. Other organs have been identified as targets, namely the lungs and kidneys [29]. The lung is the second most affected organ by the pyrroles formed after metabolic activation in the liver, since they can travel to the lungs through blood [29]. For PA to be excreted or exert toxicity, as with many xenobiotics, biotransformation must occur.

There are three principal pathways for the metabolic activation of PA, namely hydrolysis to produce necines and necic acids, *N*-oxidation to form PANO, and oxidation that leads to the formation of pyrrolic esters or dehydropyrrolizidine alkaloids (DHPA). Hydrolysis is an important detoxification route, promoting the clearance of these compounds [14], as well as *N*-oxidation, which allows the formation of PANO that can be conjugated for excretion. However, PANO can reverse back into PA and suffer oxidation into DHPA [29]. This route is carried out mostly by cytochrome P-450 (CYP450) monooxygenases. In fact, the activity of these enzymes can partly explain the distinct susceptibility of different species to PA [29]. The isoforms of CYP450 involved in the metabolism leading to DHPA are generally CYP3A and CYP2B [30]. In the case of hydrolysis, liver microsomal carboxylesterases are involved [30]. However, only retronecine-type and heliotridine-type PA are capable of suffering *N*-oxidation, otonecine-type PA being unable to generate PANO owing to their methylation in the nitrogen [14].

The balance between the formation of DHPA and the formation of detoxification compounds, such as necines, necic acids and PANO, is also important in explaining the distinct susceptibility of different species to these compounds [31].

**Figure 8:**
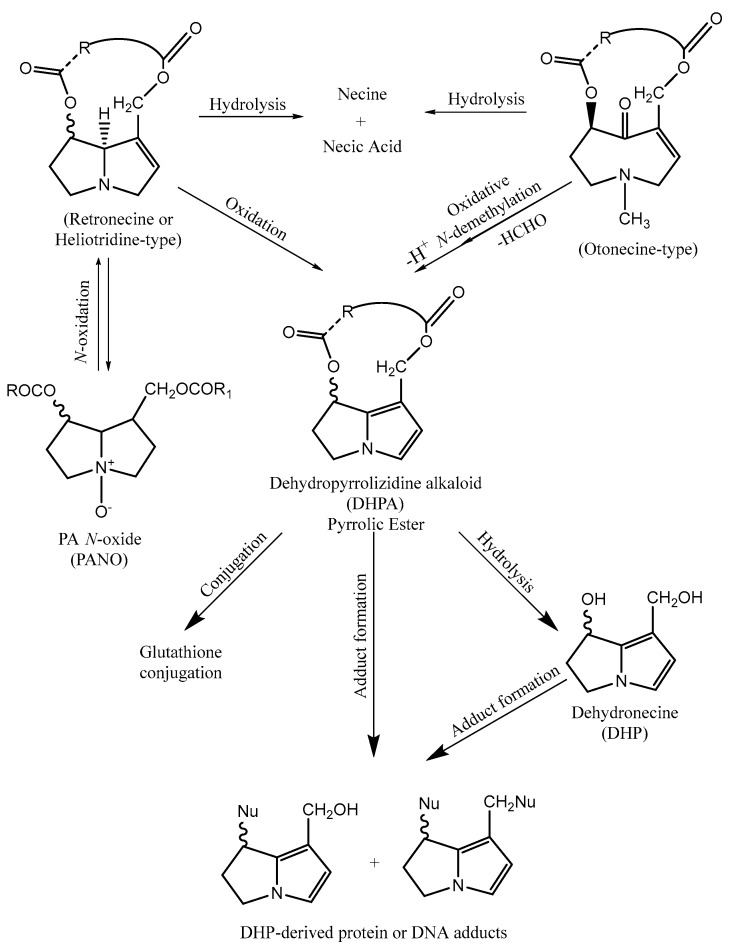
Metabolism of PA.

The formation of DHPA happens through hydroxylation of the necine base at C-3 and C-8 positions, in the specific case of retronecine- and heliotridine-types [14]. In otonecine-type, an oxidative *N*-demethylation is necessary [14]. After these highly reactive metabolites are formed, they can bind to glutathione (GSH) to form GSH conjugates and in doing so, they can be eliminated [32], which is the reason that conjugation to GSH is considered a detoxification route [32]. In the same way, pyrrolic esters can bind to proteins and deoxyribonucleic acid (DNA) and, consequently, they can form adducts. These metabolites can also suffer hydrolysis and be transformed in dehydronecines, which are also toxic metabolites, but are less reactive than the previously mentioned form [32]. Figure 8 illustrates the metabolism of PA.

### 3.2. Pharmacological Properties

Despite the toxicity described in some experimental models, which will be discussed later, PA exhibit an interesting spectrum of biological properties, which can be exploited in drug discovery programs.

#### 3.2.1. Anti-Microbial Activity

Many alkaloids have been described as effective anti-microbials, which is in line with the defensive role of this class of secondary metabolites in plants [33].

In the specific case of PA, the anti-microbial activity of usaramine, monocrotaline and azido-retronecine against some bacteria has been demonstrated [34]. Usaramine was analyzed concerning its ability to inhibit biofilm formation in *Staphylococcus epidermidis* and *Pseudomonas aeruginosa*. Although the mechanism of action of usaramine remained unclear, it was possible to observe that it prevented the formation of biofilm by *S. epidermis* by about 50% at 1 mg/mL. However, no effect was detected in the formation of biofilm by *P. aeruginosa*. Furthermore, monocrotaline and azido-retronecine demonstrated anti-*Trichomonas vaginalis* activity (concentrations up to 1 mg/mL), being lethal to 70% and 85% of bacterial cells, respectively, while was devoid of toxicity towards *T. vaginalis*. Interestingly, no detectable damage in vaginal epithelial cells was found, a selectivity trait that may be relevant for the development of new drugs, such as topic anti-microbial agents.

In another study, the effects of PA from *Senecio jacobaea* L. were investigated for their effect on the growth of nine plant-associated fungi (five strains of *Fusarium oxysporum*, two of *Fusarium sambucinum* and two of *Trichoderma* sp.) [35]. A PA mixture consisting of senecionine (12%), seneciphylline (22%), jacobine (24%) and jaconine (24%) was highly effective, however high concentrations were required, the effective range of each individual PA varying from 0.33 mM to 3.33 mM, the most sensitive fungus belonging to the *Trichoderma* genus.

#### 3.2.2. Anti-Inflammatory Activity

The inflammatory process is a physiological response of the body in order to eliminate, neutralize and/or destroy *stimuli* resulting from infection or tissue damage [36].

In inflammatory processes, the upregulation of inducible nitric oxide synthase as a consequence of pro-inflammatory mediators, such as cytokines, results in increased levels of nitric oxide (·NO), which plays an important role as a mediator in the inflammatory response [37]. Therefore, the regulation of its production in tissues may be important for the treatment of inflammation.

In a study by Huang et al., six new PA and two that were already known were isolated from *Liparis nervosa* (Thunb.) Lindl. and evaluated for their inhibitory capacity towards ·NO production by lipopolysaccharide (LPS)-challenged RAW 264.7 macrophages. The new molecules tested were nervosine I, nervosine II, nervosine III, nervosine IV, nervosine V, nervosine VI, and the previously-described PA were lindelofidine and labumine. Overall, all molecules were effective in this model, with IC_50_ values ranging from 2.16 to 38.25 μM [38].

Another study with the same cell line led to the conclusion that PA present in an ethanol extract of the plant *Heliotropium digynum* (Forssk.) C. Chr inhibited the production of ·NO by 78% at 25 μg/mL [39]. In this work, the IC_50_ values found for heliotrine, heliotrine *N*-oxide, 7-angelyolsincamidine *N*-oxide and europine were 52.4, 85.1, 105.1 and 7.9 μM, respectively.

Crotalaburnine was evaluated for its activity against increased vascular permeability and oedema induced by formaline, carrageenin, 5-hydroxytryptamine, dextran, bradykinin and prostaglandin [40]. This alkaloid was also tested against the formation of granulation tissues by cotton-pellet in rats. Its effects were compared with the activity of different compounds known for their anti-inflammatory properties, such as hydrocortisone [40]. Results showed that this PA was only efficient against acute edema induced by carrageenin and hyaluronidase, with a dose of 10 mg/kg [40]. In the cotton-pellet granuloma test it was shown that crotalaburnine was two times more potent than hydrocortisone [40].

#### 3.2.3. Anti-Cancer Activity

In 1992, researchers in the area of pediatric cancer treated 31 children with acute lymphoblastic leukemia with indicine-*N*-oxide at two dose levels (2000 mg/m^2^/day and 2500 mg/m^2^/day) for 5 consecutive days [41]. Among the 12 patients treated with 2000 mg/m^2^/day, 1 achieved a complete response after 6 months. On the other hand, of the 16 patients treated with 2500 mg/m^2^/day, 1 reached a similar response after 1 month. The patient with chronic myelogenous leukemia displayed a partial response in 4 months. These results suggested that indicine-*N*-oxide is active in the treatment of acute lymphoblastic leukemia of children. However, it has a narrow therapeutic index and a very steep dose response curve. At the doses tested, mild acute hepatotoxicity was registered. However, the administration of doses ≥3000 mg/m^2^/day for 5 days caused severe hepatotoxicity. Another study involving patients with ages between 4 and 67 years confirmed that indicine-*N*-oxide can induce remissions in cases of acute and chronic leukaemia at the concentration of 3000 mg/m^2^ administered daily for 5 days. In this study, only 1 out of 22 cycles of treatment resulted in liver failure [42].

In a study using different human cancer cell lines (cervical, breast, prostate and cervical squamous) indicine *N*-oxide from *Heliotropium indicum* L. inhibited the proliferation of the previous referred cancer cell lines, with IC_50_ values ranging from 46 to 100 μM [43]. At these concentrations, cell cycle arrest at mitosis was detected, without noticeable changes in the organization of the spindle or interphase microtubules.

#### 3.2.4. Anti-HIV Activity

Polyhydroxylated PA have been described as capable of interacting with human immunodeficiency virus (HIV) activity [44]. Australine and alexine, isolated from *Castanospermum australe* A. Cunn. & C. Fraser ex Hook and *Alexa Leiopetela* Sandwith, are examples of these polyhydroxylated PA that in concentrations between 0.1 and 10 mM inhibited, in distinct degrees, the activity of glycosidases, particularly the nitrogen-linked glycosylation process of HIV [44]. This event ultimately results in reduced cell fusion with the virions and, consequently, restricted syncytium formation [45].

A study from Taylor et al. with alexine and other four PA isolated from *A. leiopetala* and *C. australe*, respectively, also showed inhibitory activity against HIV-1 [46]. The positive results were obtained with 7,7a-diepialexine and an IC_50_ of 0.38 mM was found. This anti-HIV activity was correlated with the inhibition of pig kidney α-glucosidase 1 and the diminished cleavage of the precursor HIV-1 glycoprotein gp160.

#### 3.2.5. Acetylcholinesterase Inhibitors

Acetylcholinesterase (AchE) is an enzyme that catalyzes the hydrolysis of acetylcholine (ACh) and other esters that act as neurotransmitters [47]. It plays an important role in neural function and it is mainly present in the synaptic gaps of central and peripheral nervous system, being responsible for terminate nerve impulses [47]. Overstimulation of ACh receptors can lead to disorders like depression. However, when present in low amounts, other diseases can manifest, namely Alzheimer and *Myasthenia gravis* [47,48]. For this reason, inhibitors of this enzyme are exploited as therapeutic targets [47].

Benamar et al. isolated four PA from *Solenanthus lanatus* DC., including a new one named 7-*O*-angeloylechinatine-*N*-oxide, together with 3′-*O*-acetylheliosupine-*N*-oxide, heliosupine-*N*-oxide, and heliosupine [49]. All of these compounds inhibited AChE, with IC_50_ values between 0.53 and 0.60 mM. A more recent study, from the same author, with 7-*O*-angeloyllycopsamine-*N*-oxide, echimidine-*N*-oxide, echimidine, and 7-*O*-angeloylretronecine isolated from *Echium confusum* Coincy showed the inhibition of AChE, with IC_50_ values ranging from 0.275 to 0.769 mM [50].

#### 3.2.6. Miscellaneous

A work with the leaves and inflorescences from *Senecio brasiliensis* (Spreng.) Less., performed by Toma et al. on mice and rats, shed a light on the possible use of PA in the treatment of ulcerogenic disease and stomach pain [51]. The therapeutic doses of PA were assessed by the administration of hydrochloric acid/ethanol to induce gastric ulcer. It was possible to perceive that the extent of the lesion induced was significantly reduced by 32.9%, 42.5% and 66.8% with concentrations of 12.5, 25 and 50 mg/kg of PA extract (containing senecionine, integerrimine, retrorsine, usaramine and seneciphylline), respectively. In the same work, a dose of 12.5 mg/kg of the same PA extract was shown to ameliorate nonsteroidal anti-inflammatory drugs-induced gastric ulcer [51].

## 4. Toxicity

The toxicity of PA is largely documented [52,53], being almost exclusively associated to their metabolites.

In 1968, Mattocks introduced what is now considered the main mechanism responsible for the toxicity of PA, namely the binding of DHPA with groups containing sulphur, nitrogen and oxygen present in proteins, to form adducts, such as 2,3-dihydro-1H-pyrrolizineprotein [53], mainly in the site of formation [29]. Pyrroles can also penetrate the nucleus and react with DNA, ultimately causing DNA cross-links and DNA-protein cross-links with abnormal functions, which will be the cause of damage, mainly in the hepatocyte. They can pass to the adjacent Dissé space and into the sinusoidal lumen, where they attack sinusoidal cells [29]. The injury caused by the toxic metabolites in hepatocytes and in the walls of hepatic veins, for example, is what leads to veno-occlusive disease (VOD), called nowadays hepatic sinusoidal obstruction syndrome (HSOS) [29].

After that, several studies have been conducted to add to the knowledge of this toxicity mechanism. In a study by Lin et al., serum protein adducts were detected in a PA-induced HSOS patient for the first time [54]. The authors developed an analytical approach based on liquid chromatography-mass spectrometry (LC-MS) to study these adducts and have concluded that pyrrole-protein adducts could be potential biomarkers of PA-induced HSOS. In this specific study, the observed HSOS were confirmed to arise from the consumption of a PA-containing plant, *Gynura segetum* (L.) DC. Another study with PA-induced liver injury led to the conclusion that pyrrole-protein adducts were present in the blood of all the patients, further strengthening the case for their use as biomarkers for this kind of liver injury [55].

A study by Zhu et al. showed that these adducts can also be used as a biomarker of liver tumor formation [56]. As a result, they decided to carry a study to clarify the basic kinetics of PA-derived DNA adducts, namely their persistence in vivo. The conclusion was that they can be used to monitoring or predicting chronic liver diseases, since DHPA-derived DNA adducts have sufficient stability and persistence. In the single-dose exposure, the PA-derived DNA adducts exhibited dose-dependent linearity and persisted for up to 4 weeks. Following multiple dose treatment, they persisted more than 8 weeks. In addition, they exhibit correlation with the progression of liver damage caused. Another group achieved the same conclusion, with five hepatocarcinogenic PA (lasiocarpine, retrorsine, riddelliine, monocrotaline and heliotrine) and their corresponding PANO [57]. All of them being able to produce DNA adducts, through rat liver microsomal metabolism.

### 4.1. Acute and Chronic Intoxications

As previously mentioned, the liver is the main target of toxicity caused by PA, mainly because bioactivation occurs mostly in this organ. VOD is the clinical manifestation most frequently found, being considered a marker for PA intoxication [14]. The symptoms include vomiting, enlargement of the liver and bleeding diarrhea [14].

PA intoxication can be acute, sub-acute and chronic, each of them presenting different symptoms. Acute intoxication is characterized by hemorrhagic necrosis, hepatomegaly and ascites; in sub-acute there is a blockage of hepatic veins, which leads to HSOS (primary sinusoidal damage and parenchymal cell dysfunction [58]) [59]. Chronic PA exposure is characterized by necrosis, fibrosis, cirrhosis and proliferation of the bile duct epithelium [60,61]; liver failure and death is the highest level of this toxicity [59].

### 4.2. Genotoxicity and Tumourigenicity

In 1954, Schoental et al. discovered that retrorsine was capable of inducing tumors in experimental studies in animals [30,62]. Tumors developed in liver, lung, bladder, skin, brain, spinal cord, pancreas and gastrointestinal tract were found [25]. All PA known to have this effect belong to heliotridine-, retronecine- and otonecine-types.

The mechanism responsible for the formation of tumors was clarified by Yang et al., which established that riddelline (retronecine-type) form DNA adducts, in the form of DHPA [63]. In addition, it was also demonstrated by other authors that the levels of DNA adducts induced by DHPA were associated with the appearance of tumors, so they can be used as biomarkers of the tumourigenicity caused by PA [64]. Besides the formation of DNA adducts, these compounds can also react with proteins and trigger DNA cross-linking, sister chromatid exchange and chromosomal aberrations [9,14,65].

Furthermore, PA were associated to skin cancer, since they can lead to photosensitization in animals upon their consumption and metabolism [66]. It is thought that phylloerythrin, a porphyrin derived from the damage of chlorophyll by microorganisms present in gastrointestinal tract, passes to the circulation and is excreted by the liver into the bile. However, a PA-damaged liver is unable to eliminate phylloerythrin, resulting in its accumulation in the blood and skin. In this case, when phylloerythrin is exposed to sunlight, the resulting metabolites can cause oxidative stress and lipid peroxidation in skin tissues and ultimately trigger the formation of tumors [66].

We were unable to find any reports of cancer cases in humans as a direct consequence of PA consumption. However, it has been shown before that the metabolism of riddelline in human liver microsomes is similar to that of rodents, including the formation of DNA adducts [67]. Since this PA induces liver tumors in rodents via formation of DNA adducts, it is plausible to conclude that this PA may also be genotoxic and tumorigenic to humans [67]. In fact, the National Toxicology Program in the United States has declared that riddelliine is “*reasonably anticipated to be a human carcinogen*” [68]. 

The potential role of PA in diseases such as cancer, pulmonary hypertension, congenital anomalies and liver diseases has been reviewed before [69]. These alkaloids are genotoxic and can slowly initiate diseases of this sort, which is problematic because clinicians are unware of PA dietary exposure. The authors defined six indicators that can suggest a dietary dehydroPA etiology, appointing, for example: “cirrhosis, especially if associated with HSOS and/or accumulation of copper in the liver” and “cancers and/or congenital anomalies where there is evidence of overt or asymptomatic HSOS, pulmonary arterial hypertension (PAH), bone deformities, or immunological deficiencies”. If several of these indicators are present, the authors affirm that it is possible that a dietary exposure to PA is involved in the disease etiology.

### 4.3. Other Types of Toxicity

Lungs can also be a target of injury, since DHPA can travel from the liver into pulmonary arterioles, producing damage similar to the VOD [70]. After reaching this organ, thrombi in vessels and thickening in their walls leads to occlusion and inflammation [29]. Overall, the combination of these phenomena ultimately trigger pulmonary hypertension and subsequent congestive heart failure [29]. In a study by Culvenor et al. carried out on hooded Wistar rats, it was demonstrated that PA can elicit lung lesions, as result from low-level (0.025 mmoles/kg body weight) and long-term exposure to PA [71]. Two types of lung lesions were observed: intravascular accumulation of mononuclear cells ultimately resulting in venous occlusion, and extravascular alteration, in which the alveolar septa were thickened, and the number of cells increased. The authors also concluded that rats developing lung lesions always presented chronic liver lesions.

Neurotoxicity was also reported as a part of the poisoning by these substances, particularly by tricodesmine, including symptoms like encephalitis, characterized by vertigo and headaches, which could progress to delirium and loss of consciousness [70]. At the central nervous system, necrotic lesions have been described [72].

There are also reports of teratogenicity in the literature, justified by the fact that some PA can pass the placenta, as referred above. For example, a case of hepatic VOD in a newborn of a woman who consumed herbal tea prepared from *Tussilago farfara* L. was described [73]. Also, in Australia, the consumption of *Senecio madagascariensis* Poir. by a mare was reported to lead to hepatic failure in a foal of two months [74].

A study with clivorine isolated from *Ligularia hodgsonii* Hook, in concentrations between 10 and 100 µM, showed that this PA can induce DNA fragmentation, compatible with apoptosis, in human foetal hepatocyte line and mouse hepatocytes, with IC_50_ of 40.8 µM [75,76].

### 4.4. Chemical and Biological Aspects That Influence the Toxicological Profile

The structural basis for the toxicological effects of PA have been described in some works. The presence of the 1,2 double bond, as found in retronecine-, heliotridine- and otonecine-types, has been associated with the toxic effects of PA [52], as well as the presence of one or two hydroxyl groups attached to the pyrrole ring [29]. Several studies also suggest that the presence of a methyl group at C-1 is relevant, as is the presence of two esterified groups and branching in at least one of the carboxylic acids [29]. For this reason, PA that exert the highest toxicity are cyclic diesters, monoesters being the ones that cause the lowest level of injuriousness; between them are the open-chain diesters, which cause an intermediary toxicity [25]. The existence of relationship between the esterification level and the toxicity has been suggested, as, for example, macrocyclic DHPA were revealed to be more toxic than open chain diesters [28].

Toxicity of PA can be influenced by age and gender, since members of masculine sex are a group of risk, as well as children and fetuses, which are the most vulnerable group [59]. There are also toxicological differences between distinct PA within a species and of the same PA in different species [77].

PA poisoning is exacerbated with bacteria and metals. A study from Yee et al. showed that the simultaneous exposure to low doses of monocrotaline, which would not normally cause damage, and LPS elicited hepatotoxicity [78]. In this case, centrilobular and midzonal liver lesions were registered. Aston et al. studied the impact of a copper-rich diet in PA toxicity [79]. The results showed that retrorsine and copper together led to a more serious liver damage than retrorsine alone, a result that was confirmed in another work [80].

## 5. Human and Animal Consumption of PA

### 5.1. Legal Framework

With the increasing consumption of herbal medicines, PA poisoning has begun to be regarded as a public health problem. Consequently, some countries established regulations about PA in foodstuff. In the United States of America, the Food and Drug Administration ordered the ban of all PA-containing comfrey preparations from the market [81]. The German Federal Department of Health restricted the use of these preparations to 6 weeks and in a level of less than 1 µg/day; if the use was prolonged in time, the daily limit should be reduced to 0.1 µg [82]. Another regulation implemented was the labeling of these products with the following statement: “*Not to be used in pregnancy and during the lactation period*.”, due to the susceptibility of fetuses and children to diseases instigated by PA [82,83]. In the European Union, the European Food Safety Authority (EFSA) determined that the ingestion of toxic PA induces VOD and that they have carcinogenic effects in rodents [84]. In 2011, EFSA concluded that no tolerable daily intake could be established. They followed the margin of exposure (MOE) approach, a “ratio of two factors, which assesses for a given population the dose at which a small but measurable adverse effect is first observed and the level of exposure to the substance considered”. The MOE defined was of 1:10,000 for an exposure of 7 ng/kg of body weight per day. As an example, for a 70 kg individual, this corresponds to a daily exposure of approximately 500 ng of PA [85]. The European Medicines Agency, based on toxicological considerations and the available guidelines for assessment/management of genotoxic carcinogens, also showed concern about the hazards of PA, recommending a maximum daily intake of 0.35 µg PA/day for a person with a body weight of 50 kg and life-long exposure [86]. Austria excluded all products with PA from the market, and in the Netherlands, all foodstuff, herbal preparations, and extracts of plants known to have PA were limited to 1 µg/kg or 1 µg/L in the ending product [87].

Risk assessments of PA are based on animal studies and, for this reason, different approaches were suggested to translate animal doses to human exposure risks. Guidance documents have been developed taking into account differences between species that can influence the toxicity, namely the metabolic pathways [88,89]. Some groups reviewed the relevance of animal models to predict the effects of PA in humans [90]. The findings highlight that direct comparison between animal and human results is not always possible. For example, the PA-induced tumourigenicity previously reported for animals has not, to this day, been demonstrated in humans. Anyway, it is still an open question whether the differences between species should exclude the results in animals for quantitative risk assessment in humans [91].

As extracted from the conclusions drawn by the several risk assessment authorities, there is no consensus in the PA daily intake limit, although they all concur that PA are a class of undesirable compounds in food. For this reason, quality control of foodstuffs is pivotal and can be important for establishing legally binding limits. The first step should be the choice of an appropriate and universal analytical method for PA, as it was requested by EMA to the European Pharmacopeia [91]. As far as we could determine, this is being undertaken at the moment [92].

### 5.2. Data from Literature

Due to the presence of PA in several species relevant for human and animal nutrition, they may pose a threat to human health through their presence in herbal teas, herbal medicines, dietary supplements, vegetables, cereals, wheat grain, honey and pollen [93,94,95,96,97,98]. Cases of intoxication by contaminated cereals, teas, and salads have been extensively reported [97,99,100].

In 1903, it was recognized by Gilruth that tansy ragwort (*S. jacobaea*) produced chronic liver disease in cattle [101,102]. Afterwards, in 1956, a study by Bull and Dick showed that species from *Crotalaria* spp. led to comparable diseases [103]. A serious outbreak with the consumption of bread made from wheat contaminated with seeds of *Heliotropium* sp. plants, which contain PA, happened in Afghanistan, in 1974–1975 [104]. The patients exhibited ascites and emaciation, typical of hepatic VOD. Equally, in Tajikistan (1993), an epidemic was observed involving wheat contaminated by *Heliotropium lasiocarpum* Fisch. & C.A.Mey. As consequence, 3906 cases of liver diseases were registered, leading to over 60 deaths [105].

In 1989, the International Program on Chemical Safety, an agency of the World Health Organization and Food and Agriculture Organization, published the “Pyrrolizidine Alkaloids Health and Safety Guide” [83]. This guide contained statements about the hazards for humans and animals and the confirmation that contaminated grain, herbal medicines, beverages, foodstuff or grazing with PA could cause acute or chronic illness [83].

PA poisoning was initially a problem, mainly in developing countries, as result of the use of traditional medicines containing PA (Table 1) [100]. However, in the last years, there has been a growing focus on this type of medicine in industrialized countries, thus making this problem a wider concern [100]. In Europe, chronic toxicity due to long-term consumption of food or herbal medicines containing these alkaloids is now a reality [106].

Several studies on food chemistry and food safety have shown that many of the foodstuff currently consumed are sources of this type of alkaloids. A recent study from Mulder et al. showed that the contamination of eggs and meat products with PA seems to be rare in the European Union [124]. Nevertheless, PA are sometimes found in milk, albeit in very low concentrations, since milk suffers from extensive processing, during which these compounds are diluted [124]. The class of PA found in milk revealed that *Senecio* spp. and species from the Boraginaceae family could be the origin of their occurrence [124].

In the last few years it has been reported that even herbal teas and teas not prepared from plants known to have PA in their composition, such as *M. chamomilla* and *Mentha × piperita* L., can have high amounts of these compounds, as consequence of cross-contamination [107,125]. When studying the distribution of PA in herbal teas, namely green, black, peppermint, rooibos, chamomile and one mix of herbs, it was observed that the most frequent was the senecionine-type (senecionine-, retrorsine-, seneciphylline-, senecivernine-*N*-oxides and their respective free bases) [124]. Lycopsamine- and heliotrine-types were less frequently found, intermedine being the most common, followed by lycopsamine-*N*-oxide and heliotrine-*N*-oxide [124]. The highest average concentration was from senecionine-*N*-oxide (1.73 µg/L and 64% frequency), followed by retrorsine-, seneciphylline-, senecivernine-*N*-oxides and the corresponding free bases [124]. Together, these PA accounted for 76% of total PA content found in herbal teas; lycopsamine- and heliotrine-types accounted for 24%, while monocrotaline-type was not present [124]. Moreover, PANO were found in higher concentrations than the corresponding free bases. High amounts of PA were also revealed in tea, namely in black and green tea from retail market, an unexpected finding [124]. As described before, different chemical types were identified, open-chain diesters being mainly perceived in fennel (*F. vulgare*) infusion and cyclic PA in black tea [107].

Concerning food supplements, the samples analyzed were often contaminated with PA, being the amounts highly variable [124]. The analysis was made considering three types of food supplements: supplements based on plants not known to produce PA (*Valeriana officinalis* L., *Hypericum perforatum* L.), supplements based on plants known to produce PA (*B. officinalis*, *Eupatorium perfoliatum* L., *Eupatorium odoratum* L., *L. officinale*, *Pulmonaria officinalis* L., *S. officinale*, *Petasitis* sp., *P. hybridus*, *T. farfara*), supplements containing bee products (pollen, propolis and royal jelly). Food supplements made from plant material known for their content in PA revealed the highest PA levels, those from lycopsamine-type (lycopsamine, intermedine, echimidine) being the more common [124]. However, the supplements made from plants not known to produce PA similarly demonstrated to have these compounds, probably due to cross-contamination. Supplements made of oil-based extracts of PA-producing plants were devoid of PA, whereas the presence of PA in supplements containing bee products was also confirmed [124].

Several studies have shown that the distribution of PA subclasses varies with the vegetal material [126]. For example, while pollen is richer in seneciphylline-type, in flower heads retrorsine- and usaramine-types are more common [126].

Among the several food products that can harbor these toxins, honey is one of the most studied and important [126,127,128]. PA have been found in honey from various botanical and geographical origins [129]. Senecionine, echimidine and lycopsamine, in particular, were present in *Echium* spp. honey samples coming from Spain [130]. Considering the concentrations found by Kempf et al. in honey samples (0.019–0.120 µg/g) and that a common dose is 1 or 2 table spoons per day (10–20 g), it is possible to conclude that a honey consumer can easily exceed the recommended limit: maximum of 1.0 µg of PA per day [129,131,132,133,134,135]. A study from Lucchetti et al. [127] revealed the presence of PA in nectar from *Echium vulgare* L. Echimidine corresponded to half of the PA content found and acetylechimidine, vulgarine, echiuvulgarine and acetylvulgarine were the other half. They also concluded that pollen frequently exhibited higher levels of PA than nectar, but the proportion of the diverse types of these compounds found in honey was more closely related to that found in nectar compared to that present in pollen. For this reason, there are some doubts about the origin of these toxins in honey, since it is composed by nectar, but also contains traces of pollen.

PA-containing plants known to be used in the production of honey can be found in Table 2.

Studies with pollen from *S. vernalis* revealed that PA-free honey can be contaminated when this pollen is added to it, probably by diffusion from pollen to honey [93]. For some authors, it was also clear that pollen contained much higher levels of PA than honey and that pollen appeared in it at low doses [126]. However, a study showed the opposite, specifically that a relationship between the concentration of pollen in the honey and its PA levels is not always found, since honeys with considerable amounts of PA on their composition are revealed to have low levels of pollen [129].

## 6. Conclusions

PA are a widespread group of secondary metabolites that can, in certain situations, pose a life threat to humans and animals, once they are present in a variety of foodstuff. These compounds have became known for their toxicity, as per several outbreaks that were registered, mainly in developing countries. However, in the last years, industrialized countries began to face this reality, when the use of traditional medicines increased. Despite this, some PA can also be useful, since they demonstrate pharmacological properties which can be further exploited by relying in medicinal chemistry strategies that can maintain bioactivity while reducing toxicity.

Thereby, for the sake of human and animal health protection, it is of great importance to further develop the information regarding the chemistry, pharmacology and toxicology of PA.

## Figures and Tables

**Table 1:** Medicinal species containing PA [17,20].

Family	Plant	Reference
Apiaceae	*Foeniculum vulgare* Mill.; *Pimpinella anisum* L.; *Carum carvi* L.	[107]
Apocynaceae	*Amphineurion marginatum* (Roxb.) D. J. Middleton; *Alafia* cf. *caudata* Stapf	[108]
Asteraceae	*Eupatorium cannabinum* L.; *Adenostyles alliariae* (Gouan) Kern; *Emilia sonchifolia* (L.) DC.; *Petasites hybridus* (L.) PH Gaertn., B. Mey & Scherb.; *Petasites spurius* (Retz) RCHB; *S. jacobaea*; *Senecio vulgaris* L.; *T. farfara; Senecio nemorensis* L.; *Ageratum conyzoides* L.; *Chromolaena odorata* (L.) R. M. King & H. Rob.; *Eupatorium chinense* L.; *Eupatorium fortunei* Turcz.; *Eupatorium japonicum* Thunberg ex Murray.; *Cacalia hastata* L.; *Cacalia hupehensis* Hand.-Mazz.; *Crassocephalum crepidioides* (Benth.) S. Moore; *Farfugium japonicum* (L.) Kitam.; *Gynura bicolor* (Roxb. ex Willd.) DC.; *Gynura divaricata* (L.) DC.; *G. segetum; Ligularia dentata* (A.Gray) Hara; *Petasites japonicus* (Siebold & Zucc.) Maxim.; *Senecio argunensis* Turcz.; *Senecio integrifolius* (L.) Clairv.; *Senecio scandens* Buch.-Ham. Ex D. Don; *Syneilesis aconitifolia* (Bunge) Maxim.; *Matricaria chamomilla* L.; *Gynura pseudochina* (L.) DC.; *Gynura japonica* (Thunb.) Juel; *Packera candidissima* (Greene) W. A. Weber & Á. Löve; *Solanecio mannii* (Hook.f.) C. Jeffrey; *Solanecio tuberosus* (Sch. Bip. ex A. Rich.) C. Jeffrey var. tuberosus; *Bidens pilosa* L.; *Senecio longilobus* Benth.	[60,107,109,110,111,112,113,114,115,116,117,118]
Boraginaceae	*Alkanna tinctoria* (L.) Tausch; *Anchusa officinalis* L.; *Borago officinalis* L.; *Cynoglossum officinale* L.; *Heliotropium arborescens* L.; *Lithospermum officinale* L.; *Myosotis scorpioides* L.; *Symphytum asperum* Lepech; *Symphytum caucasicum* Bieb.; *Symphytum officinale* L.; *Symphytum tuberosum* L.; *Symphytum* × *uplandicum* Nyman; *Arnebia euchroma* (Royle) I. M. Johnst.; *Cordia myxa* L.; *Cynoglossum amabile* Stapf & J. R. Drumm; *Cynoglossum lanceolatum* Forssk.; *Cynoglossum zeylanicum* (Vahl) Brand; *Cynoglossum grande* Dougl. ex Lehm.; *Cynoglossum virginianum* L.; *Arnebia benthamii* (Wall. ex G.Don.) Johnst.; *H. indicum*; *Lappula intermedia* (Ledeb.) Popov; *Lithospermum erythrorhizon* Siebold & Zucc.	[119,120,121,122,123]
Fabaceae	*Crotalaria albida* Roth; *Crotalaria assamica* Benth.; *Crotalaria pallida* Aiton; *Crotalaria sessiliflora* L.; *Crotalaria tetragona* Andrews	/
Lamiaceae	*Melissa officinalis* L.	[107]
Orchidaceae	*L. nervosa*	/
Urticaceae	*Urtica dioica* L.	[107]

**Table 2:** Plants containing PA used in the production of honey in several countries.

Country	Plant	Reference
Argentine	*Senecio grisebachii* Baker	[136]
Australia	*Echium plantagineum* L.; *E. vulgare*; *Eucryphia lucida* (Labill) Baill; *Heliotropium amplexicaule* Vahl; *Heliotropium europaeum* L.	[131]
Brazil	*C. pallida; Eupatorium* sp.	[137,138]
Bulgaria	*T. farfara*	[139]
China	*E. plantagineum*; *E. vulgare*; *Senecio* spp.; *C. officinale; Tussilago* spp.	[140]
Ethiopia	*Solanecio angulatus* (Vahl) C. Jeffrey	[109]
Germany	*E. vulgare*; *Phalaenopsis* sp.; *S. jacobaea*; *Senecio vernalis* Waldst. & Kit.	[126]
Ghana	*C. odorata*; *Eupatorium* spp.; *Ageratum* spp.	[141]
India	*Crotalaria juncea* L.	[76,142]
Italy	*Echium* sp.; *Senecio erucifolius* L.; *Senecio inaequidens* DC; *S. jacobaea*; *S. vulgaris*; *Robinia pseudoacacia* L.	[143,144,145]
New Zealand	*E. vulgare*; *B. officinalis*; *Echium* spp.	[134,140]
Portugal	*Echium* sp.	[143]
South Africa	*S. inaequidens*; *Senecio pterophorus* DC	[143,145]
Spain	*E. plantagineum*; *E. vulgare*	[135]
Switzerland	*E.vulgare*; *Eupatorium* sp.; *Senecio* sp.	[140,146]
Thailand	*E. odoratum*	[147]
Turkey	*Myosotis* sp.	[148]
United Kingdom	*Borago* sp.; *S. jacobaea*	[140,149]
United States	*C. officinale*; *E. vulgare*; *S. jacobaea*; *S. vulgaris*; *S. officinale*	[150,151]
Uruguay	*E. plantagineum*	[152]

## Abbreviations

AChE	Acetylcholinesterase
ACh	Acetylcholine
CYP 450	Cytochrome P450
DHPA	Dehydropyrrolizidine alkaloid(s)
DNA	Deoxyribonucleic acid
EFSA	European Food Safety Authority
GSH	Glutathione
HSOS	Hepatic sinusoidal obstruction syndrome
HIV	Human immunodeficiency virus
LPS	Lipopolysaccharide
MOE	Margin of exposure
·NO	Radical nitric oxide
PA	Pyrrolizidine alkaloid(s)
PANO	Pyrrolizidine alkaloids *N*-oxide(s)
VOD	Veno-occlusive disease
